# Does regulation of skeletal muscle function involve circulating microRNAs?

**DOI:** 10.3389/fphys.2014.00039

**Published:** 2014-02-17

**Authors:** Wataru Aoi, Kunihiro Sakuma

**Affiliations:** ^1^Laboratory of Health Science, Graduate School of Life and Environmental Sciences, Kyoto Prefectural UniversityKyoto, Japan; ^2^Health Science Center, Toyohashi University of TechnologyToyohashi, Japan

**Keywords:** microRNA, skeletal muscle, circulation, exosome, exercise, muscular disease

## Abstract

MicroRNAs (miRNAs) are small non-coding RNAs involved in post-transcriptional gene regulation. Recently, growing evidence has shown that miRNAs are taken in by intracellular exosomes, secreted into circulation, and taken up by other cells. Circulating levels of several miRNAs are changed in diseases such as cancer, diabetes, and cardiovascular diseases; therefore, they are suggested to regulate functions of the recipient cells by modulating protein expression. Circulating miRNAs (c-miRNAs) may also modulate skeletal muscle function in physiological and pathological conditions. It has been suggested that acute and chronic exercise transiently or adaptively changes the level of c-miRNAs, thus post-transcriptionally regulating proteins associated with energy metabolism, myogenesis, and angiogenesis. Circulating levels of several miRNAs that are enriched in muscle are altered in muscle disorders and may be involved in their development and progression. In addition, such c-miRNAs may be useful as biomarkers to determine various interactions between tissues and also to reflect athletic performance, physical fatigue, incidence risk, and development of diseases.

## Introduction

**Figure 1:**
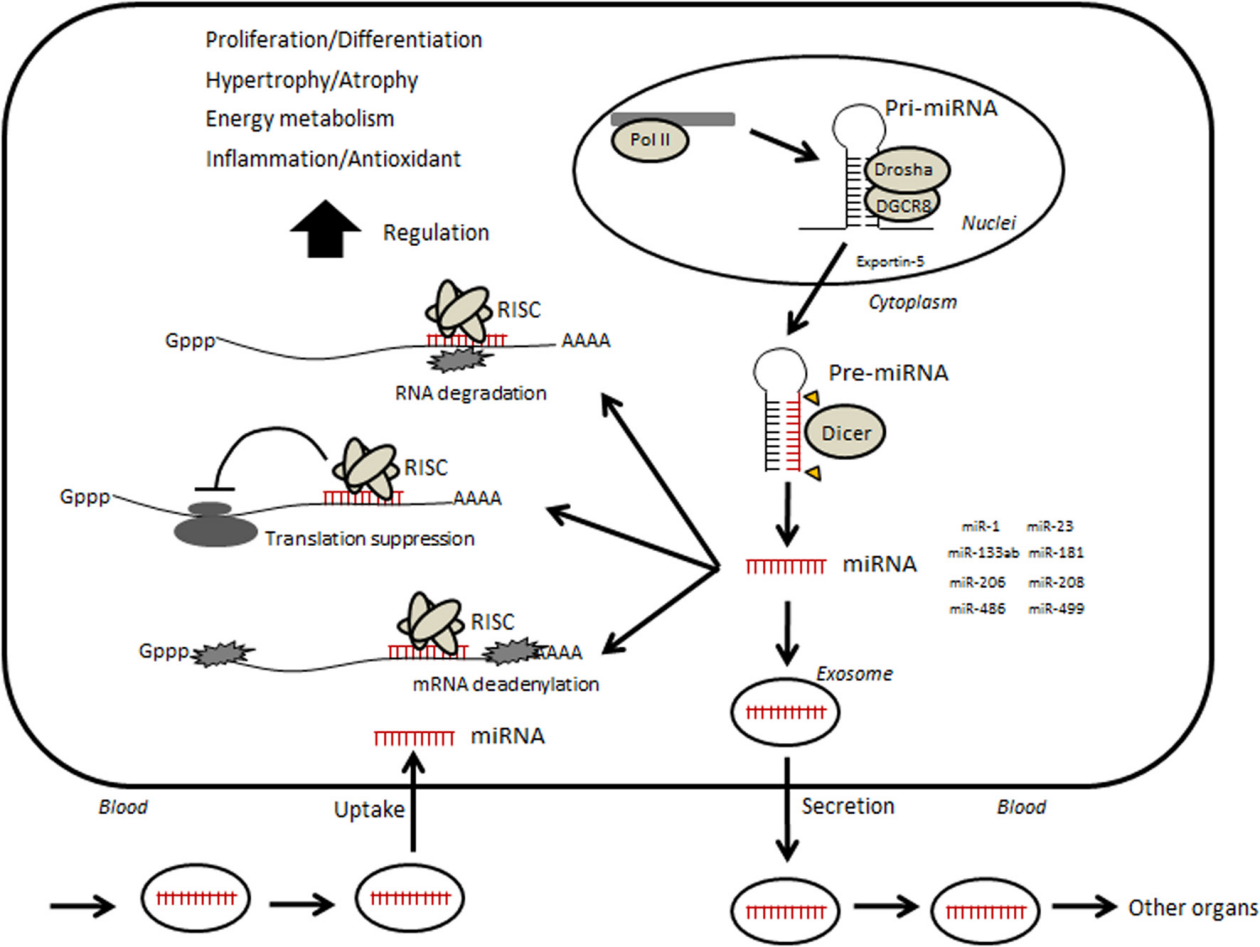
**Hypothetical illustration of relevance between circulating microRNA and skeletal muscle**. Small non-coding RNAs are transcribed by RNA polymerase II (Pol II)-producing long primary transcripts (pri-miRNAs), which are then cleaved into stem-loop pre-miRNAs by the microprocessor, which includes the RNase III enzyme Drosha and DiGeorge syndrome critical region gene 8 (DGCR8). The pre-miRNA is subsequently transported from the nucleus to the cytoplasm by Exportin-5 and further cleaved by the enzyme Dicer into mature miRNAs. Mature miRNAs are incorporated into RNA-induced silencing complex (RISC). The RISC acts by hybridizing, either perfectly or partially, to complementary binding sites located in the 3′ untranslated region (UTR) of target mRNAs and inhibiting translation by mRNA cleavage or steric hindrance, which leads to various phenotypic changes. In addition, several miRNAs can be taken into an intracellular exosome and secreted into circulation. Circulating miRNAs move into other organs or muscle itself and may regulate their functions.

MicroRNAs (miRNAs) are small non-coding RNAs of approximately 19–22 nucleotides in length that regulate gene expression at the post-transcriptional level through mechanisms such as translational inhibition or mRNA degradation. DNA encoding small non-coding RNAs is transcribed by RNA polymerase II, producing long primary transcripts (pri-miRNAs) that are then cleaved into 60–70-bp stem-loop precursors (pre-miRNAs) by the microprocessor, which includes RNase III enzyme Drosha and DiGeorge syndrome critical region gene 8 (DGCR8) (Figure 1) (Kim et al., 2009; Winter et al., 2009). The pre-miRNA is subsequently transported from the nucleus to the cytoplasm by exportin-5 and further cleaved by the Dicer complex into mature miRNAs (e.g., miRNA duplex). One strand of the mature miRNAs is then incorporated into a ribonucleoprotein complex called the miRNA-induced silencing complex (miRISC) and the other strand is degraded (Wienholds and Plasterk, 2005). miRISC suppresses gene expression through hybridizing, either completely or partially, to complementary binding sites located in the 3′ untranslated region (UTR) of target mRNAs and degrading the mRNA molecules or inhibits their translation in mammalian cells (Bartel, 2004; Djuranovic et al., 2012; Pasquinelli, 2012).

Biogenesis of miRNA is regulated through several steps. First, transcriptional regulation modulates the expression of miRNA. Several genome-wide approaches have revealed transcription factors associated with miRNA promoters. Transcription factors that regulate specific miRNAs are often themselves targets of those miRNAs in positive or negative feedback loops (Martinez et al., 2008). Conversion from pri-miRNA to pre-miRNA, regulated by microprocessors, is another rate-limiting step. Microprocessor activity can be regulated by factors such as p68 and p72 (Gregory et al., 2004). Thereafter, export of pre-miRNA from the nucleus to the cytoplasm depends on the activity of exportin 5. Processing of pre-miRNA to mature miRNA is regulated by Dicer activity. In addition, the degradation rate of miRNA, e.g., stabilization, plays an important role in modulating miRNA levels.

Generally, a single miRNA can regulate the expression of over 100 mRNAs and proteins (Ambros, 2004; Bartel, 2004). In fact, over 60% of protein-coding genes may be regulated by miRNAs (Friedman et al., 2009), and much of these miRNAs are suggested to have a role in a wide range of biological processes such as development, homeostasis maintenance, and adaptation. Misexpression of miRNAs results in the onset of diseases such as immune diseases, cardiovascular diseases (CVDs), neurological diseases, and cancer (Mendell and Olson, 2012). Several miRNAs are also suggested to act as modulators of myogenesis, hypertrophy, and nutrient metabolism in skeletal muscle. Some of them mediate muscle adaptations in response to exercise and diet, as well as muscular pathogenesis. In addition, it has been known that several miRNAs are secreted from cells into circulation or taken from circulation into cells (Figure 1). Many researchers studying areas such as disease, aging, and physical exercise are now focusing on the association of c-miRNAs with various physiological and pathological phenotypes.

## Role of miRNAs in muscular function

A tissue-specific miRNA is defined as a mature miRNA that is expressed in a specific tissue at levels that are >20-fold higher than the mean levels in all other tissues (Lee et al., 2008). Recently, there has been increasing evidence regarding the roles and regulation of miRNAs in skeletal muscle. miR-1, miR-133, and miR-206 are highly enriched in both human and mouse skeletal muscle (Sempere et al., 2004). It has been shown that the expression of miR-1, miR-133a, miR-133b, and miR-206 corresponds to nearly 25% of all miRNA expression in skeletal muscle, and thus they are often referred to as muscle-specific miRNAs or myomiRs (McCarthy, 2008). These four miRNAs belong to the miR-1 family, which can be divided into two groups: miR-1/206 and miR-133a/b, on the basis of the sequence of the seed region. Thus, the regulation of these muscle-specific miRNAs as well as their relationship with muscle functions such as myogenesis, hypertrophy, and energy metabolism, has been of particular interest.

Expression of muscle-specific proteins/miRNAs is regulated by key myogenic regulatory factors (MRFs), myocyte enhancer factor 2 (MEF2), serum response factor (SRF), and myocardin-related transcription factor-A (Chen et al., 2006; Rao et al., 2006; Rosenberg et al., 2006; Liu et al., 2007; Small et al., 2010). In addition to downstream MRFs, muscle-specific miRNAs influence myoblast proliferation and differentiation through repression of SRF, histone deacetylase 4 (Chen et al., 2006), DNA polymerase (Kim et al., 2006), and the upstream paired box protein Pax-7 (Dey et al., 2010). Thus, miRNAs are involved in myogenesis via their regulatory relationship with MRFs. In addition, these miRNAs also modulate muscle hypertrophy and atrophy by acting as repressors of growth factor gene targets, chaperones, and caspases (Clop et al., 2006; McCarthy et al., 2007; Xu et al., 2007).

In addition to miR-1, miR-133a/b, and miR-206, other miRNAs such as miR-208, miR-486, and miR-499 are also abundant in muscle and have specific functional roles in skeletal muscles. miR-208b and miR-499 are encoded in introns in the myosin heavy chain genes *MHC7* and *MHC7b*, respectively, which are enriched in type I fibers (McCarthy, 2011), by regulating target genes such as Sry-box 6 (*Sox6*), thyroid hormone receptor associated protein 1 (*Thrap1*), and purine-rich element-binding protein and also by repressing β-MHC expression (van Rooij et al., 2009; Bell et al., 2010; McCarthy, 2011). In addition, another predicted target of miR-208b and miR-499 is GDF8, which is also known as myostatin, a major negative regulator of muscle mass (Drummond et al., 2009). Phosphatase and tensin homolog (PTEN) and forkhead box transcription factor O1 A, which serve as negative components of phosphoinositide-3-kinase (PI3K)/AKT signaling (Small et al., 2010), are targets of miR-486. Through gain- and loss-of-function experiments, it was shown that miR-486 modulates PI3K/AKT signaling by directly targeting PTEN, an inhibitor of PI3K phosphorylation, thereby promoting the phosphorylation of Akt and the activity of downstream components of the pathway. Furthermore, miR-181 is upregulated upon myogenic differentiation, and it targets homeobox AII, a repressor of the differentiation process, to allow new muscle growth (Naguibneva et al., 2006). miR-24 is also related to myogenesis via modulation of transforming growth factor β, myogenin, and MEF2 (Sun et al., 2008). Furthermore, some miRNAs have been suggested to regulate metabolic modulators. Increased expression of miR-696 in skeletal myocytes leads to negative regulation of the peroxisome proliferator-activated receptor gamma, coactivator 1 alpha (PGC-1α) protein, along with reduced expression of the mRNAs for its downstream genes (Aoi et al., 2010). miR-23 can also negatively regulate *PGC-1*α mRNA and protein and its downstream metabolic proteins in mice skeletal muscle (Safdar et al., 2009).

Each miRNA has many targets, making it difficult to determine the significance of miRNA changes to various physiological and pathological conditions. In addition, changes in miRNAs are often contradictory, suggesting that the effect of the miRNA and its network of gene targets on muscle phenotypic change is complex, and it is currently unclear whether we can use miRNA levels as a biomarker for particular phenotypes. Furthermore, miRNA responses likely vary between species and under different exercise and diet conditions, as well as sampling conditions.

## c-miRNAs and muscle pathology

Recently, growing evidence has shown that some miRNAs exist in circulation (Figure 1). In 2007, Valadi et al. demonstrated that miRNAs were taken into intracellular vesicles exosomes, which are small membranous vesicles derived from the endosome (Raposo and Stoorvogel, 2013), and released into circulation. Circulating miRNAs can originate from various types of cells, including parenchymal cells, blood vessel cells, and blood cells. Although a detailed mechanism for the secretion of miRNAs from cells has not yet been established, Kosaka et al. (2010b) reported that miRNA secretion was regulated by neutral sphingomyelinase 2, a rate limiting enzyme in the biosynthesis of ceramide, which triggers the secretion of exosomes. Many subsequent studies have shown the existence of circulating miRNAs (c-miRNAs) in various human body fluids, including serum, plasma, breast milk, urine, saliva, and other interstitial fluids (Kosaka et al., 2010a). In addition to exosomes, other extracellular vesicles such as microvesicles and apoptotic bodies, and non-vesicle-associated proteins such as HDL/LDL or RNA-binding proteins such as Argonaute are known to be involved in the process (Arroyo et al., 2011; Turchinovich et al., 2011; Vickers et al., 2011). Profiles of c-miRNAs are changed by conditions such as disease and pregnancy (Chim et al., 2008), indicating that c-miRNAs can be used as biomarkers to monitor such conditions (Lawrie et al., 2008). A unique characteristic of c-miRNAs is that they can circulate in the blood without degradation by RNases. In addition, c-miRNAs can be transported from circulation into other cells and can regulate functions of the recipient cells (Valadi et al., 2007). Thus, miRNAs are thought to be able to determine various interactions between tissues and to reflect physiological and pathological states.

Previously, many c-miRNAs have been identified, particularly in cancer studies. For example, miR-21 is a well-characterized miRNA that contributes to the development of cancer (Schetter et al., 2008; Medina et al., 2010). This miRNA has been shown to regulate several tumor suppressor genes (Meng et al., 2007; Asangani et al., 2008). Several reports have shown an increased expression of circulating miR-21 in the serum of patients with various types of cancer, including diffuse large B-cell lymphoma, osteosarcoma, colorectal cancer, hepatocellular carcinoma, gastric cancer, prostate cancer, and glioblastoma (Lawrie et al., 2008; Skog et al., 2008; Yaman Agaoglu et al., 2011; Zhou et al., 2011; Kanaan et al., 2012; Li et al., 2012; Ouyang et al., 2013); therefore, for diagnosis, it may be useful to examine the expression of circulating miR-21 in the serum of cancer patients. Likewise, circulating levels of miRNAs such as miR-1 and miR-133, which are highly expressed in the heart, are increased in CVDs. Because these miRNAs are more abundant in the serum of patients than that of normal/healthy subjects, they might be useful as potential biomarkers for disease. Indeed, c-miRNAs may contribute to the pathogenesis of disease by regulating protein expression in target cells (Mitchell et al., 2008; Wang et al., 2009, 2010; Heneghan et al., 2010). In culture, secreted miRNA is transferred into a recipient cell, where it exerts its function (Kosaka et al., 2010b). However, it is unclear how circulating miRNAs are associated with disease onset, development, or recovery.

As mentioned above, several miRNAs act as modulators of skeletal muscle cell function such as proliferation, differentiation, hypertrophy, and nutrient metabolism (Chen et al., 2006; Cardinali et al., 2009; McCarthy et al., 2009; Dey et al., 2010). Indeed, muscle disorders, exercise, immobilization, and intake of amino acids can change the level of miRNAs in skeletal muscle, which is suggested to account for phenotypic changes (Eisenberg et al., 2007; McCarthy et al., 2007; Safdar et al., 2009; Aoi et al., 2010; Nielsen et al., 2010; Deng et al., 2011; Mizuno et al., 2011). Although the mechanisms underlying the secretion of skeletal muscle miRNAs into blood by particular physiological and pathological conditions remain unclear, many researchers focus on defining c-miRNA profiles, which may include a possibility to become useful biomarkers for such conditions and may be involved in translating muscle phenotypes to whole body phenotypes, e.g., muscle disorder and exercise-induced health promotion (Table 1).

**Table 1:** **Change of circulating microRNAs in muscular physiological and pathological conditions**.

**Condition**	**Increase**	**Decrease**	**References**
Dystrophy	miR-1, miR-133ab, miR-206		Cacchiarelli et al., 2011; Mizuno et al., 2011; Roberts et al., 2013; Vignier et al., 2013
COPD	miR-1, miR-206, miR-499		Donaldson et al., 2013
Rhabdomyosarcoma	miR-1, miR-133ab, miR-206		Miyachi et al., 2010
Type 2 diabetes	miR-144		Karolina et al., 2011
Acute aerobic exercise	miR-1, miR-133ab, miR-21, miR-126, miR-146a, miR-181a, miR-208b, miR-221, miR-222	miR-9, miR-23ab, miR-31, miR-486	Baggish et al., 2011; Uhlemann et al., 2012; Aoi et al., 2013; Banzet et al., 2013; Russell et al., 2013
Acute resistance exercise	miR-149^*^		Sawada et al., 2013
Aerobic exercise training	miR-20a	miR-486	Baggish et al., 2011; Aoi et al., 2013
High fitness level		miR-21, miR-210, miR-222	Bye et al., 2013

COPD, chronic obstructive pulmonary disease.

Recently, it has been reported, in animal studies, that several miRNAs that are highly expressed in muscle can be detected in plasma and serum, and are changed by muscle disorders. Mizuno et al. (2011) have shown that the serum levels of miR-1, miR-133a, and miR-206 are increased in Duchenne muscular dystrophy (DMD) models, dystrophin-deficient muscular dystrophy mouse (*mdx*) and X-linked muscular dystrophy dog, compared with normal animals. Although intramuscular proteins such as creatine kinase, myoglobin, and lactate dehydrogenase are generally known as classic circulating biomarkers of muscle disorders, it is lower specificity to determine presence/absence of disease because its level is easily elevated by physical stress, such as intense exercise (Vassella et al., 1965; Nicholson et al., 1986). In contrast, the levels of these c-miRNAs are much less affected by physical stress compared with the levels of intramuscular proteins (Mizuno et al., 2011), suggesting that the circulating levels of these muscle-specific miRNAs may be more useful and reliable biomarkers for muscular dystrophy. Thereafter, Roberts et al. (2013) confirmed that the dystrophy-involved miRNAs (miR-1, miR-133a, and miR-206) in serum show dynamic patterns of expression with the progression of muscle pathology in *mdx* mice and that these changes of c-miRNAs are significantly correlated with the levels of regeneration factors such as myogenin in muscle tissue, suggesting these c-miRNAs may be used as biomarkers of muscle turnover (i.e., myofiber degeneration and regeneration) although this must be experimentally validated. The upregulation of miR-1, miR-133a, miR-133b, and miR-206 also has been confirmed in human DMD patients compared with age-matched subjects (Cacchiarelli et al., 2011; Vignier et al., 2013), which is in agreement with the results from animal models (Mizuno et al., 2011; Roberts et al., 2013). In addition to being higher in dystrophic disorders, levels of muscle-specific miRNAs, including miR-1, miR-206, and miR-499, are higher in plasma of patients with chronic obstructive pulmonary disease, who often exhibit reduced muscle fiber size and proportions due to mechanisms such as inflammation-induced protein catabolism, compared with control subjects (Donaldson et al., 2013). In addition, serum levels of muscle-specific miRNAs (miR-1, miR-133a, miR-133b, and miR-206) are significantly higher in patients with rhabdomyosarcoma tumor than in those with non-rhabdomyosarcoma tumors (Miyachi et al., 2010). These results suggest that a change in the level of these c-miRNAs may be useful as a biomarker for the clinical diagnosis of rhabdomyosarcoma in the future, for which there is no serum biomarker currently known. Furthermore, Karolina et al. (2011) found that the level of circulating miR-144 increased in type 2 diabetic animals and humans and that this elevation is negatively correlated with insulin receptor substrate 1 in insulin-responding tissues, including skeletal muscle. Thus, the elevation of miR-144 in circulation may be associated with the development of insulin resistance in skeletal muscle.

## c-miRNA and physical exercise

Baggish et al. (2011) were the first to show that exercise affects the levels of c-miRNAs associated with angiogenesis and inflammation in competitive male rowers: a single bout of exhaustive cycling or rowing training for 90 days elevated miR-20a, miR-21, miR-146a, miR-221, and miR-222 levels in plasma. Although the sources of exercise-induced c-miRNAs remain unclear, a variety of tissue types relevant to exercise (muscles, vascular endothelium, and plasma-based platelets and leukocytes) can release c-miRNA into the extracellular space, including plasma. In addition, positive correlations between the peak levels of miR-146a and VO_2max_ and between changes in the ratio of resting miR-20a to VO_2max_ from pre-training to post-training have been reported (Baggish et al., 2011). Therefore, changes in c-miRNAs may be fitness biomarkers and physiological mediators of exercise-induced cardiovascular adaptation although this must be experimentally validated. Recently, Bye et al. (2013) assessed whether c-miRNAs are associated with VO_2max_-level in healthy individuals. They found that miR-21, miR-210, and miR-222 were higher in the low VO_2max_-group than in the control group. There were no correlations between traditional risk factors for CVD (blood pressure, cholesterol, smoking habit, or obesity) and miR-21, miR-210, and miR-222; however, the authors suggested that these miRNAs have a potential as new, independent biomarkers of fitness level and risk of future CVD.

Recently, we investigated the effect of acute and chronic exercise on typical muscle-specific miRNAs in serum obtained from young healthy subjects who were not habituated to a regular exercise regimen. We found that almost all muscle-specific miRNAs (miR-1, miR-133a, miR-133b, miR-206, miR-208b, and miR-499) were present at very low levels in serum (Aoi et al., 2013), in accordance with the results reported by other groups (Baggish et al., 2011; Mizuno et al., 2011), suggesting their low secretion from muscle cells into circulation in healthy humans. In contrast, we reported that a single bout of cycling exercise at 70% VO_2max_ for 60 min decreased the circulating levels of the muscle-specific miRNA miR-486 immediately after the exercise (Aoi et al., 2013). This decrease in circulating miR-486 was also found in the resting state following 4 weeks of cycling training at 3 days per week. In addition, the change ratio of miR-486 due to acute exercise showed a significant negative correlation with VO_2max_ for each subject. One mechanism to explain the decrease in circulating miR-486 in response to exercise may be reduction in secretion of the miRNA from muscle cells. However, although the half-life of miR-486 is unknown, 60 min of exercise is probably too short a period to cause a decrease in circulating levels by reducing secretion. Another possibility is that exercise may accelerate the uptake of circulating miR-486 into certain recipient cells. Several studies (Valadi et al., 2007; Kosaka et al., 2010b; Mittelbrunn et al., 2011) have shown through *in vitro* experiments that miRNAs contained in exosomes are transferable from outer media into recipient cells, where they affect their biological functions. Previously, we found that miR-486 levels tended to increase in skeletal muscle after exercise training in mice (data not shown); this may partly be caused by uptake from circulation to muscle cells although we must experimentally confirm that circulating mi-486 is transferable and functional in the muscle cells. A major putative target of miR-486 is PTEN, which is a negative regulator of phosphoinositide-3-kinase/Akt signaling, a major pathway downstream of the insulin receptor (Small et al., 2010). It is well-known that a single bout of exercise activates insulin signaling in muscle cells, thus contributing to glucose uptake as an energy substrate of muscle contraction. miR-486 may conduct glucose uptake via activation of insulin signaling and suppression of PTEN, which helps maintain muscle contraction during exercise. Thus, miR-486 regulates insulin-dependent glucose uptake in metabolic tissues such as the skeletal muscle, and this may be associated with the negative correlation between circulating miR-486 and VO_2max_.

In contrast to previous studies showing that most muscle-specific circulating miRNAs are unchanged by exercise, Banzet et al. (2013) reported that miR-1, miR-133a, miR-133b, and miR-208b can be changed depending on the exercise mode. They show that these miRNAs were not affected by uphill walking (concentric), but significantly increased during early recovery of downhill waking (eccentric) (2–6 h) in healthy subjects. The resulting elevation of muscle-specific miRNAs may be caused by cellular leakage with muscle damage following eccentric exercise (muscle-damaging exercise), which was confirmed by Uhlemann et al. (2012). However, Russell et al. (2013) showed that in the 3-h period following a single bout of cycling (non-muscle-damaging exercise), miR-1, miR-133a, miR-133-b, and miR-181a levels were increased in untrained male subjects. Therefore, low levels of circulating muscle-specific miRNAs can be also secreted in response to exercise. Interestingly, they also reported an elevation in the miRNA biogenesis pathway (Drosha, Dicer, and exportin-5), and a reduction in the levels of muscle myopathy-related miRNAs (miR-9, miR-23a, miR-23b, and miR-31) by acute exercise (Russell et al., 2013), which may be associated with exercise-derived improvement of muscle function. In addition to endurance exercise, Sawada et al. (2013) investigated the c-miRNA profile that is affected by acute resistance exercise. Twelve healthy subjects performed a resistance exercise session (bench press and leg press), consisting of five sets of 10 repetitions at 70% of maximum strength. However, they could not find any significant changes regarding muscle-specific miRNA in serum, regardless of muscle-damaging exercise protocol. Instead, the level of miR-149^*^ in serum increased on the day following resistance exercise, and miR-146a and miR-221 levels decreased 3 days after exercise. However, the significance of these changes has not been yet been clarified, because there is no correlation with other circulating parameters related to muscle building.

Regular exercise can improve skeletal muscle function, including nutrient metabolism and muscle strength, along with reducing the risk of CVD, type 2 diabetes, and cancer. In addition to the adaptive effects provided by regular exercise, even a single bout of exercise induces various benefits including metabolic improvement. Although the detailed mechanism remains unknown, c-miRNAs, as well as other circulating factors, e.g., hormones, adipokines, and myokines, may be associated with exercise-induced benefits.

## Perspectives

The amount of research on miRNAs has grown drastically in the past 10 years, once we understood that non-coding RNA could affect phenotypes via post-translational regulation. In addition, in the past 5 years, there was a breakthrough in research when it was suggested that miRNAs are present in extracellular fluids and are changed by various pathological and physiological events. In the field of skeletal muscle research, such circulating miRNAs are expected to be found to underlie cellular mechanisms and become biomarkers for athletic performance, physical fatigue, and incidence risk and development of diseases. However, no miRNAs have been established as such biomarkers for use in the fitness or clinical fields.

To advance research and translation, we must address a number of issues. First, the mechanism of miRNA secretion from supply cells and uptake into recipient cells must be characterized. Indeed, the transfer system of some miRNAs has been reported in culture experiments (Valadi et al., 2007; Kosaka et al., 2010b; Mittelbrunn et al., 2011), but c-miRNAs altered by muscle disorders and physical exercise have not been described. The c-miRNA binding proteins in the exosome and other extracellular vesicles have not been identified. In addition, we must determine how c-miRNAs recognize their receptor cells. Second, we must develop protocols for quantification of c-miRNA. The results of preliminary microarray profiles are often different from those obtained by quantitative PCR (Chen et al., 2009; Sato et al., 2009), perhaps due to the difficulty in designing specific probes and primers for miRNAs, which are short and highly similar. It is also important to develop sampling techniques to reduce hemolysis in normal body fluid (such as by optimizing needle thickness) and to identify the ideal fluid sample (e.g., plasma or serum) (McDonald et al., 2011; Wang et al., 2012). The problem of how to normalize c-miRNA levels is also important, because it is unclear whether an extracellular housekeeping gene can be used for this purpose. Further research is needed to characterize the detailed mechanisms and physiological and pathological relevance of changes in c-miRNAs, and must be based on appropriate measurement protocols.

### Conflict of interest statement

The authors declare that the research was conducted in the absence of any commercial or financial relationships that could be construed as a potential conflict of interest.
